# The correlation between strength and range of motion of the neck muscles and opium smoking in Iran

**DOI:** 10.3389/fpsyt.2023.1200091

**Published:** 2023-05-30

**Authors:** Omid Massah, Amir Masoud Arab, Ali Farhoudian, Mehdi Noroozi, Fahimeh Hashemirad

**Affiliations:** ^1^Substance Abuse and Dependence Research Center, University of Social Welfare and Rehabilitation Sciences, Tehran, Iran; ^2^Department of Physiotherapy, University of Social Welfare and Rehabilitation Sciences, Tehran, Iran; ^3^Department of Psychiatry, Tehran University of Medical Sciences, Tehran, Iran; ^4^Department of Psychiatry, University of Social Welfare and Rehabilitation Sciences, Tehran, Iran; ^5^Psychosis Research Center, University of Social Welfare and Rehabilitation Sciences, Tehran, Iran

**Keywords:** opium smoking, drug use disorder, neck range of motion, neck muscles strength, Iran

## Abstract

**Objectives:**

Opium smoking is commonly practiced via traditional and novel routes in Iran. Both smoking methods are practiced in a non-ergonomic position. According to previous studies and our hypothesis, it can be potentially harmful to the cervical spine. Thus, the present study aimed to investigate the relationship between opium smoking and neck range of motion and neck muscle strength.

**Methods:**

In this cross-sectional and correlational study, the range of motion and strength of the neck muscles of 120 men with drug use disorder were measured by a CROM goniometer and a hand-held dynamometer. Other data gathering was performed using a demographic questionnaire, the Maudsley Addiction Profile, and the Persian version of Leeds Dependence Questionnaire. The obtained data were analyzed by Shapiro–Wilks test, Pearson’s correlation coefficient and stepwise linear regression.

**Results:**

There was no significant correlation between the age of drug use onset and range of motion and muscle strength of the neck; however, the daily duration of opium smoking and the number of years of opium smoking were inversely and significantly correlated with the range of motion and muscle strength of the neck in some directions. Daily opium smoking time for decreasing in neck range of motion and total duration of opium smoking for reduction of neck muscles strength are stronger predictor variables.

**Conclusion:**

Opium smoking by traditional routes causes non-ergonomic positions and has a moderate significant correlation with reduced range of motion and neck muscle strength, in Iran.

**Highlights:**

– The harm of drug use disorder is not only AIDS and hepatitis, and harm reduction programs should go beyond the prevention of AIDS and hepatitis. According to more than 90% of smoking use of drug compared to other methods (oral and injectable, etc.) musculoskeletal disorders caused by the smoking use of drugs, have a greater cost burden in reducing the quality of life and the need for rehabilitation.
– Drug abuse treatment and harm reduction programs should focus more seriously on replacing smoking use of drugs with oral medications assisted treatment.
– Although in Iran and some countries in the region, a large number of people smoke opium for many years and sometimes all their lives, daily in a completely non-ergonomic position, but studying the deformation of the posture and musculoskeletal disorders related to the body position in them, is not a scientific concern and neither physical therapy researchers have paid attention to it nor addiction researchers.
– Neck muscles strength and range of motion in opium addicts are correlated to the number of years of opium smoking and daily minutes of opium smoking, but not to its oral use.
– There is no significant correlation between the onset age of continues and permanent opium smoking and substance dependence severity with neck range of motion and muscles strength.
– People with drug use disorder (especially smoking users) as a large group of vulnerable people, should be the target population of musculoskeletal disorders researchers and addiction harm reduction researchers, and more experimental, comparative, cohort, etc. researches should be designed and implemented for them.

## Introduction

The physical posture of individuals is generated by their movement habits. Moreover, it is formed on a morphological and functional basis and is a manifestation of the individual’s physical and mental conditions (1). Therefore, it is an indicator of one’s kinetic status, as well as muscle balance and neuromuscular coordination. The human body condition is influenced by changes throughout life. In fact, posture visibly echoes musculoskeletal activity (2, 3). Modern urban life is associated with rapid changes in the environment, lifestyle, physical activity restrictions, and improper nutrition (4). Despite numerous preventive and protective measures, many health problems are caused by the contemporary world’s lifestyle (5).

As long as corrective action is not taken to improve posture, its adverse effects on the body will continue and postural pressure will be imposed on the person. Accordingly, the odds of musculoskeletal disorders related to work or non-ergonomic position remains high (3). Performing repetitive tasks in incorrect posture and non-neutral position leads to postural strain, fatigue, and pain (6). In such positions, the muscles bear further load and are exposed to damage along with the surrounding connective tissues. If continued and repetitive, these positions can lead to irreversible changes in the form of shortening or stretching of muscle fibers and soft tissues (advancing from the elasticity stage to the plasticity phase) (7, 8). Therefore, being in certain recurrent positions over days and hours leads to alternations in the musculoskeletal system. In addition, such cases have been proven in various operations and sports activities (9, 10). Considering the high prevalence of musculoskeletal disorders in today’s societies (11), investigating the prevalence of these conditions and the factors affecting them in diverse age groups, genders, occupations, etc., is an essential area of research in rehabilitation. The smoking of opium is common in traditional (using a device called Vafour) and novel (using a type of hookah called in Iran “Gholgholei” or using spoke and pin called in Iran “Sikh-o-sang”) methods in Iran. Furthermore, as both methods are a non-ergonomic position, individuals with opium use disorder who consume via smoking route are more prone to some musculoskeletal disorders. This issue can be attributed to long-term exposure to non-ergonomic positions during consumption, as well as the lack of movement, malnutrition, and unhygienic conditions, heavy smoking, and so on (12–14). Opium smoking is not common in other regions. In European and American countries, heroin and marijuana are mainly smoked, and their smoking time is very short, compared to opium; therefore, this issue has not been a research priority in Europe and America. Opium smoking is very common in the Middle East and especially in Iran (15). In addition, its use has increased after the onset of Coronavirus Disease 2019 (COVID-19) pandemic (due to the false belief that opium use can prevent the spread of COVID-19) (16). Iran has the highest prevalence rate of opium smoking in proportion to the population globally (17), Thus, this study aimed to investigate the relationship between the Range of Motion (ROM) of the neck and its muscle strength and opium smoking and the severity of opium use disorder.

## Methods

This cross-sectional and correlation study was conducted in 2021 in Tehran City, Iran. By snowball sampling method, we selected 120 men from four main branches according to the inclusion and exclusion criteria of the study among the referrals to four outpatient and residential substance abuse treatment centers.

The inclusion criteria were as follows: a diagnosis of substance use disorder according to the International Classification of Diseases-11th Revision (ICD-11) criteria (18), ability to stand, age between 25 and 50 years, Body Mass Index (BMI) below 27.5 kg/m^2^ (there are lower probabilities of musculoskeletal disorders in these age and BMI ranges). Also, the exclusion criteria were a history of neuromuscular or skeletal disease, a history of surgery in spine and shoulder girdle areas, a history of championship or practicing sports regularly, any impairments in balance control caused by a specific disease, any obvious postural deformities and anatomical disorders, and using smartphones and tablets for more than 30 min a day (19).

Data collection was performed using demographic questionnaire, Maudsley Addiction Profile (MAP) (20) and Persian version of Leeds Dependence Questionnaire (LDQ) (21). The maximal isometric strength of flexor, extensor, and lateral flexor muscles of the neck (MVIC) was measured by a hand-held dynamometer (Model: Micro Manual Muscle Tester; North Coast Medical Inc.). Before starting the measurement, the device was calibrated using standard weights. The dynamometer was programmed for 7 s, and after placing the device on the head, the person increased the contraction force of the muscle within 2 s to reach the maximum contraction force, and then kept it in the same position for 5 s. All tests were performed once (due to the odds of encountering fatigue in repetitions and associations distorted results) and the data obtained from the strength measurement tests were normalized to the BMI of the study subjects. The neck ROM was calculated using a cervical inclinometer (Model: Baseline CROM-3 goniometer), while the subject was sitting on a chair and the chest was tied to the chair support with a tight band.

To confirm the reliability of the measurement method, 10 subjects participated in an extra four testing sessions of neck muscle strength and ROM test in 2 weeks.

Statistical analysis was done by SPSS software version 23 through Shapiro–Wilk test, correlation coefficient tests (ANOVA and Pearson) and stepwise linear regression.

The study has been approved by the Ethics Committee of the University of Social Welfare and Rehabilitation Sciences with the code of IR.USWR.REC.1398.120. This article is extracted from doctoral thesis of the first author.

## Results

The mean ± SD age of the study participants was 39.30 ± 5.05 years and their mean BMI score was 24.29 ± 2.12 kg/m^2^. 53 people were workers, 58 people were employees, and the rest were unemployed. Also, 37 people had primary education, 68 people had high school education, seven people had university education, and the rest were illiterate. The substance use profile of the study participants is available in Table 1.

**Table 1:** Substance use profile of participants.

Variables	Mean	SD
Age of substance use onset (year)	23.15	7.20
Age of continued substance use (year)	28.20	6.15
The duration of any type of substance use (year)	16.50	8.25
The duration of opium smoking (month)	110.45	31.70
Daily opium smoking time (minute)	212.35	48.45
The severity score of substance use disorder	25.20	4.35

The results of inter-rater and intra-rater reliability tests suggested that the measurement methods were reliable. For inter-rater reliability, the Intraclass Correlation Coefficients (ICCs) ranged from 0.6 (CI: 0.18–0.86) for measuring the range of left lateral flexion to 0.88 (CI: 0.64–0.95) for forward flexion; regarding muscle strength, ICCs ranged from 0.64 (CI: 0.22–0.91) for extension to 0.92 (CI: 0.66–0.97) for forward flexion. For intra-rater reliability, the ICCs ranged from 0.68 (CI: 0.20–0.90) for right lateral flexion to 0.94 (CI: 0.86–0.98) for extension; and in muscle strength ICCs, they ranged from 0.68 (CI: 0.20–0.89) for left lateral flexion to 0.9 (CI: 0.65–0.93) for extension.

There was no significant relationship between the onset age of permanent opium smoking and the ROM of the neck. However, a significant correlation was recorded between the opium smoking duration (months/lifetime) and daily opium smoking time (minutes/day), and the ROM of the neck in most directions (Table 2).

**Table 2:** Correlation coefficients of neck range of motion with the age of starting permanent opium smoking, drug dependence severity, daily opium smoking duration, and life opium smoking duration.

Variables		Correlation coefficient	*p* value
Predictor variable	Criterion variable		
Job^1^	Forward flexion ROM	0.38^*^	0.045
	Extension ROM	0.15	>0.05
	Right lateral flexion ROM	0.13	>0.05
	Left lateral flexion ROM	0.19	>0.05
	Right lateral rotation ROM	0.24	>0.05
	Left lateral rotation ROM	0.29	>0.05
Consumption route^1^	Forward FLEXION ROM	0.23	>0.05
	Extension ROM	0.38	>0.05
	Right lateral flexion ROM	0.17	>0.05
	Left lateral flexion ROM	0.16	>0.05
	Right lateral rotation ROM	0.31	>0.05
	Left lateral rotation ROM	0.18	>0.05
The onset age of continues and permanent opium smoking (year)	Forward flexion ROM	0.19	0.112
	Extension ROM	0.23	0.209
	Right lateral flexion ROM	0.22	0.095
	Left lateral flexion ROM	0.31	0.092
	Right lateral rotation ROM	0.33	0.117
	Left lateral rotation ROM	0.24	0.213
Total duration of substance use	Forward flexion ROM	0.11	0.296
	Extension ROM	0.32	0.098
	Right lateral flexion ROM	0.29	0.114
	Left lateral flexion ROM	0.34	0.132
	Right lateral rotation ROM	0.28	0.337
	Left lateral rotation ROM	0.36	0.105
Opium smoking duration (month)	Forward flexion ROM	−0.36	0.065
	Extension ROM	−0.63^*^	0.022
	Right lateral flexion ROM	−0.59^**^	<0.001
	Left lateral flexion ROM	−0.39	0.055
	Right lateral rotation ROM	−0.52^*^	0.001
	Left lateral rotation ROM	−0.43^*^	0.040
Daily opium smoking time (minute)	Forward flexion ROM	−0.22	0.096
	Extension ROM	−0.61^**^	<0.001
	Right lateral flexion ROM	−0.33	0.068
	Left lateral flexion ROM	−0.24	0.135
	Right lateral rotation ROM	−0.46^*^	0.040
	Left lateral rotation ROM	−0.63^**^	<0.001
Drug dependence severity	Forward flexion ROM	0.11	0.092
	Extension ROM	0.24	0.174
	Right lateral flexion ROM	0.27	0.090
	Left lateral flexion ROM	0.30	0.102
	Right lateral rotation ROM	0.19	0.103
	Left lateral rotation ROM	0.29	0.078

1 The exact probability value for nominal variables was mentioned only if it was significant; otherwise, >0.05 was written.

*p < 0.05, **p < 0.001

Furthermore, as per Table 3, the maximum contraction strength of the neck muscles were significantly and inversely correlated with the opium smoking duration (months/lifetime) and daily opium smoking duration (minutes/day).

**Table 3:** Correlation coefficients of neck muscles strength with the age of starting permanent opium smoking, drug dependence severity, daily opium smoking duration and life opium smoking duration.

Variables		Correlation coefficient	*p* value
Predictor variable	Criterion variable		
Job	Flexor muscles strength	0.39	>0.05
	Extensor muscles strength	0.27	>0.05
	Right lateral flexor muscles strength	0.21	>0.05
	Left lateral flexor muscles strength	0.14	>0.05
Consumption route	Flexor muscles strength	0.20	>0.05
	Extensor muscles strength	0.16	>0.05
	Right lateral flexor muscles strength	0.11	>0.05
	Left lateral flexor muscles strength	0.22	>0.05
Onset age of continues drug use (year)	Flexor muscles strength	0.21	0.112
	Extensor muscles strength	0.18	0.214
	Right lateral flexor muscles strength	0.26	0.108
	Left lateral flexor muscles strength	0.24	0.091
Total duration of substance use	Flexor muscles strength	0.33^*^	0.038
	Extensor muscles strength	0.37^*^	0.049
	Right lateral flexor muscles strength	0.31	0.174
	Left lateral flexor muscles strength	0.33	0.094
Opium smoking duration (month)	Flexor muscles strength	−0.33^*^	0.044
	Extensor muscles strength	−0.35^*^	0.037
	Right lateral flexor muscles strength	−0.41^*^	0.046
	Left lateral flexor muscles strength	−0.39^*^	0.021
Daily opium smoking time (minute)	Flexor muscles strength	−0.29^*^	0.036
	Extensor muscles strength	−0.51^*^	0.007
	Right lateral flexor muscles strength	−0.33^*^	0.018
	Left lateral flexor muscles strength	−0.44^*^	0.005
Drug dependence severity	Flexor muscles strength	0.33	0.203
	Extensor muscles strength	0.24	0.088
	Right lateral flexor muscles strength	0.29	0.085
	Left lateral flexor muscles strength	0.19	0.105

*p < 0.05, **p < 0.001

In order to determine the most effective independent variable in decreasing the range of motion and muscles strength of neck, stepwise regression analysis was used. For this purpose, the average of the total range of motion of the neck in all six directions was calculated for each participant and considered as a unique index of the range of motion of the neck. Also, average muscle strength in four directions was used as an index of neck muscle strength in regression analysis. As shown in Table 4, opium smoking duration (month) is the first predictive variable for the possibility of decreased neck muscles strength and daily opium smoking time (minute) for decrease in neck range of motion. According to the results of the regression analysis, daily opium smoking time predicts the reduction of neck range of motion. According to these results, it predicts 10% of decreasing the range of motion of neck and the sum of the two predictive variables (daily opium smoking time and opium smoking duration) predicts 16%. 13% of the decrease in the strength of the neck muscles can be predicted by the duration of opium smoking and 17% by the sum of the opium smoking duration and daily opium smoking time.

**Table 4:** Stepwise regression analysis for neck range of motion and neck muscles strength (criterion variable) based on predictor variables.

Criterion variable	Step	Predictor variable	*R*	*R^2^*	Adjusted *R*^2^	*β*	*p value*
Neck range of motion	1	Daily opium smoking time (minutes)	0.393	0.154	0.101	0.39	<0.001
	2	Opium smoking duration (month)	0.459	0.210	0.160	0.20	<0.001
Neck muscles strength	1	Opium smoking duration (month)	0.425	0.181	0.130	0.42	<0.001
	2	Daily opium smoking time (minutes)	0.474	0.225	0.176	0.23	<0.001

## Discussion

Perhaps in recent decades, the most serious complication and the riskiest consequence of substance use disorder is the transmission of Human Immunodeficiency Virus (HIV) and hepatitis viruses to substance users, through injection drug use. However, it is certainly not the most frequent issue, especially if we consider the harms associated with substance use disorder regionally and the most common route of substance use in that region (17). For example, in Iran, on the one hand, the use of injectable drugs was greatly reduced by starting an opioid maintenance treatment program with agonist medications (22, 23). On the other hand, the use of smoking drugs, especially opium smoking, is traditionally and historically very common (15). Moreover, its prevalence increased due to the COVID-19 pandemic (16). Therefore, the harm of substance use disorder is no longer limited to HIV and Hepatitis contradiction. Thus, depending on the specific geographical region, the scope of harm reduction programs should be expanded. Musculoskeletal-related adverse effects of substance smoking, i.e., caused by long-term placement in non-ergonomic positions, are prevalent disorders. Subsequently, if not taken into account in prevention and harm reduction programs, such conditions can exorbitant rehabilitation costs.

Many people, depending on their habit or work needs, foster an inappropriate body position, which causes postural pains in the long term. Each individual, depending on the type of practiced physical or sports activities, is prone to certain types of mild postural abnormalities or deviations, i.e., suitable for that activity at that given time (24, 25). However, over years of repetition, these postural deviations may lead to a wide range of disorders (26). This is why disabled people who use a computer head controller have reduced neck ROM and neck muscles strength in some directions, which correlates with their head movement habits (27). Obviously, the unusual neck movements and non-ergonomic positions performed during opium smoking are related and consistent with the opium smoking duration (minutes per day) and the years of overall course of opium smoking. In this study, there was an inverse and significant correlation between the ROM and muscles strength of the neck in most directions with the increase in the duration of opium smoking. To some extent, these findings are in line with those of Ghamkhar’s study. In their study, although the performance of the neck muscles did not reveal a relationship with forward head posture and even neck pain and disability (in patients with chronic neck pain), the reduction in endurance and strength of the neck muscles was associated with some postural deformities (28). These findings are also in line with the results of studies conducted on dentists. Because dentists also work in the same harmful position for several hours a day, and the reduction of function and mobility of the neck and the strength of the neck muscles and decreasing in neck range of motion has been proven in many studies (29–31).

Posture deformities, i.e., mostly acquired and caused by non-ergonomic positions, are related to the ROM of the neck (32, 33). As per the study of Quek et al., there was a significant correlation between thoracic hyperkyphosis and forward head posture, and the ROM of the neck (34); these results are in line with those obtained in this study. A large body of literature has reported a relationship between repetitive work positions and the use of upper body and upper limbs when operating with work tools, and the occurrence of musculoskeletal disorders of the neck (35). Opium smoking tools also force the user to hold this position for several hours a day. As a result, after years, the risk of such conditions, as reduced ROM of the neck and declined strength of the neck muscles is not far from expected. In a similar way and with the same mechanism, welders and many workers in other industries suffer from reduced neck range of motion (36, 37). Decreasing in neck muscles strength due to non-ergonomic positions of opium smoking is similar to what happens in industries workers (38).

Furthermore, previous studies indicated a correlation between psychosocial characteristics and mental health confounders as well as the incidence of musculoskeletal disorders; the results of this study are in line with these prior investigations. This is because substance use disorder is among the main psychosocial health disorders in today’s societies (39, 40).

## Limitations and suggestions

The main limitation of this study was the lack of previous similar studies and the literature review was not very helpful. I hope this study will draw the attention of researchers and policymakers to the allocation of funds and efforts for this issue, and will make them document more convincing results with more extensive studies and with more accurate scientific and experimental methods. Another limitation of this study was that the samples were only male. Although we did not intend to do so from the beginning, we had to use only men.

## Conclusion

The traditional and novel methods of opium smoking, which are used in the Persian Gulf countries, the Middle East, Central Asia, and even the countries of East Asia, and require sitting in non-ergonomic positions for long hours, have a significant correlation with neck problems and it seems to be effective in reducing muscle strength and range of motion of neck.

## Data availability statement

The raw data supporting the conclusions of this article will be made available by the authors, without undue reservation.

## Ethics statement

The studies involving human participants were reviewed and approved by the Ethics Committee of the University of Social Welfare and Rehabilitation Sciences with the code of IR.USWR.REC.1398.120. This article is extracted from doctoral thesis of the OM in PhD by Research of Addiction Studies. The patients/participants provided their written informed consent to participate in this study.

## Author contributions

Data gathering and data analysis were done by OM. OM, AA, AF, MN, and FH contributed to design, drafting, and writing and editing of the article. All authors contributed to the article and approved the submitted version.

## Conflict of interest

The authors declare that the research was conducted in the absence of any commercial or financial relationships that could be construed as a potential conflict of interest.

## Publisher’s note

All claims expressed in this article are solely those of the authors and do not necessarily represent those of their affiliated organizations, or those of the publisher, the editors and the reviewers. Any product that may be evaluated in this article, or claim that may be made by its manufacturer, is not guaranteed or endorsed by the publisher.
